# VIP Regulates the Development & Proliferation of Treg *in vivo *in spleen

**DOI:** 10.1186/1710-1492-7-19

**Published:** 2011-11-29

**Authors:** Anthony M Szema, Sayyed A Hamidi, Marc G Golightly, Todd P Rueb, John J Chen

**Affiliations:** 1 Stony Brook University School of Medicine, Department of Medicine, 100 Nicolls Road, Stony Brook, NY 11794, USA; 2 Stony Brook University School of Medicine, Department of Pathology, 100 Nicolls Road, Stony Brook, NY 11794, USA; 3 Stony Brook University School of Medicine, Department of Preventive Medicine, 100 Nicolls Road, Stony Brook, NY 11794, USA; 4 Veterans Affairs Medical Center, 79 Middleville Road, Northport, NY, 11768, USA

## Abstract

**Background:**

Mounting evidence supports a key role for VIP as an anti-inflammatory agent and promoter of immune tolerance. It suppresses TNF-α and other inflammatory cytokines and chemokines, upregulates anti-inflammatory IL-10, and promotes immune tolerant cells called T regulatory (Treg) cells. VIP KO mice have recently been demonstrated to have spontaneous airway and pulmonary perivascular inflammatory responses, as part of asthma-like and pulmonary hypertension phenotypes, respectively. Both inflammatory responses are correctable with VIP. Focusing on this model, we have now investigated the influence of VIP not only on inflammatory cells but also on Treg cells.

**Methods:**

Using flow cytometric analysis, we examined the relative preponderance of CD25+CD4+ cells and anti-inflammatory Treg cells, in extracts of thymus and spleen from VIP KO mice (5 VIP KO; 5 VIP KO+ VIP; 10 wild-type). This method allowed antibody-based flow cytometric identification of Treg cells using surface markers CD25 and CD4, along with the: 1) intracellular activation marker FoxP3; and 2) Helios, which distinguishes cells of thymic versus splenic derivation.

**Conclusions:**

Deletion of the VIP gene results in: 1) CD25+CD4- cell accumulation in the thymus, which is corrected by VIP treatment; 2) more Treg in thymus lacking Foxp3 expression, suggesting VIP is necessary for immune tolerance; and, 3) a tendency towards deficiency of Treg cells in the spleen, which is normalized by VIP treatment. Treg lacking Helios are induced by VIP intrasplenically rather than by migration from the thymus. These results confirm the dual role of VIP as an anti-inflammatory and immune tolerance-promoting agent.

## Introduction

### Background

We hypothesized: 1) Vasoactive Intestinal Peptide (VIP) may be regulating the development and proliferation of regulatory T lymphocytes (Treg); and 2) VIP can efficiently and quickly induce Treg. Because they promote immune tolerance and are anti-allergic, Treg are important. Current methods of allergy immunotherapy, for tree pollen, for example, reduce seasonal symptoms and medication usage and costs, but are not efficient to induce Treg and require slow protocols, taking years to reach maximal dosage. The underlying immune mechanisms of VIP and Treg interactions are not entirely known. Better understanding of the VIP-Treg system may pave the way to use VIP as an adjunct or replacement for allergy immunotherapy, a treatment for allergic asthma.

A defect in current literature is that other investigators have studied *in vitro *immune responses to VIP but lacked the *in vivo *VIP knockout mouse model. Knowing that Treg are a critical cell type to engage in order to induce tolerance in allergic individuals, and having the availability of VIP knockout (VIP KO) mice--a spontaneous model of asthma (airway inflammation and airway hyper-responsiveness not requiring allergic sensitization)--we were uniquely positioned to validate the role of VIP in Treg expression from central thymus and peripheral spleen in the VIP KO mice, untreated and treated with exogenous VIP replacement.

We also studied VIP KO mice under conditions of allergic challenge and discovered large dendritic cell accumulation, suggesting an immature dendritic cell phenotype.

Vasoactive Intestinal Peptide (VIP) is a neuropeptide with properties not only as a vasodilator and smooth muscle relaxant, as originally discovered by Sami I. Said and Victor Mutt [1], but also has potent anti-inflammatory effects. VIP is present in a variety of cells, including mast cells and lymphocytes. VIP induces the release of the anti-inflammatory cytokine IL-10 and suppresses TNF-α and pro-inflammatory cytokines IL-2, IL-4, IL-5, IL-6, IL-12, IL-17, chemokines GRO/KC, and CCL5 [2-9]. In recent literature, VIP also improves immune tolerance by increasing anti-inflammatory, immune-tolerant T regulatory (Treg) cells in spleen [10].

We showed earlier that mice lacking the gene for VIP have spontaneous features of asthma, with airway inflammation (peribronchiolar lymphocytes and eosinophils) and pro-inflammatory cytokine production in bronchoalveolar lavage fluid--yielding IL-5 and IL-6 [11]. It is unnecessary to use allergic sensitization to induce these asthmatic changes, making the VIP mouse model a unique genetic asthma phenotype. When VIP KO mice are treated with VIP, these aspects of inflammation are attenuated. Another characteristic of VIP KO mice is lymphocytic perivascular inflammation of pulmonary arteries. VIP treatment attenuates these features [2]. One treatment of allergic asthma is allergy immunotherapy to induce immune tolerance by increasing Treg to allergens, which are antigens such as tree pollen. Specific injection immunotherapy with dilute doses of tree pollen often entails a slow course of two and a half years, making this an inefficient process.

Delgado *et al*. reported that VIP treatment of dendritic cells renders them anti-inflammatory and tolerogenic. These VIP-treated dendritic cells induce T cells to produce anti-inflammatory cytokine IL-10, and these T cells have low proliferative capacity, indicating immune suppression or tolerance. They also found that these VIP-treated dendritic cells, when stimulated with lipopolysaccharide, are antigen-specific. In addition, a slight increase in FoxP3 mRNA expression was found in CD4+ T cells generated with tolerogenic dendritic cells. FoxP3 is necessary for survival and function of regulatory T cells (Treg)--key cells in maintaining tolerance [10,12]. The critical role of Treg in preventing autoimmune disease and maintaining immune tolerance is well established [13,14]. Prasse found that inhaled VIP in sarcoidosis patients led to bronchoalveolar lavage fluid (BALF) Treg cell populations expressing FoxP3. This was concurrent with significantly reduced production of inflammatory cytokine TNF-α by cells isolated from BALF [15].

The Helios transcription factor has recently been described as a central thymic transcription factor in regulatory T cells independent of FoxP3 [16]. The immunoregulatory role of VIP-Helios interactions has not been studied.

With the use of VIP KO mice we demonstrated that VIP replacement attenuated the asthma phenotype but had not yet delineated a VIP's mechanism of action [2]. This model now offers the opportunity to sample Treg from thymus and spleen and test the hypotheses that: 1) VIP may be regulating the development and proliferation of Treg; and 2) VIP may be efficient and quick in induction of Treg.

## Materials and methods

VIP knockout (KO) mice, backcrossed to C57BL/6, were prepared as described [17]. We bred the mice locally and genotyped them to confirm the absence of the VIP gene [17]. We mated homozygous KO males with homozygous KO females or, if necessary, with heterozygous KO females. For genotyping, we extracted DNA from 1-cm-long tail snips using a DNA isolation kit (Qiagen, Valencia, CA). DNA (100 ng) was subjected to PCR using primers to detect both VIP and the neomycin cassette. Control, wild-type (WT) C57BL/6 mice were from Taconic Laboratories (Germantown, NY). All experiments and animal care procedures were approved by the Institutional Animal Care and Use Committee and were conducted according to National Institutes of Health *Guide for the Care and Use of Laboratory Animals*.

Experiments were performed on 3 groups of mice (n = 5 in VIPKO and VIPKO+VIP groups; n = 10 in the wild-type group): 1) 6-9 month-old C57BL/6 control male mice; 2) age and gender-matched VIP KO mice treated with buffer PBS; and 3) VIP KO mice treated with VIP at 15 nmol *i.p*. every other day for 2 weeks (7 doses). VIP knockout mice have been described previously (reference). One day after the last dose of VIP or buffer, the mice were anesthetized with pentobarbital 10 mg/kg, tracheotomized and 0.8 ml of blood were withdrawn in a heparinized tube via cardiac puncture. The spleen and thymus were removed and placed in a Falcon tube with RPMI media and stored on ice until labeling with antibodies for flow cytometric analysis of FOXP3 and Helios. The spleen and thymus were teased apart and disaggregated on top of a cell strainer in a cell culture dish containing 5 ml of cold media (FACS wash). The cell suspension was transferred to a 50 ml conical tube, centrifuged 10 minutes, resuspended in 5 ml RBC lysis buffer, and incubated for 3 minutes at room temperature. The cells were then centrifuged and washed twice with 15 ml FACS wash buffer, 350 × g 5 minutes and resuspended at 5 × 10^6^/ml.

The cells were labeled with 5 microliter One step staining Mouse Treg Flow™ kit (FoxP3 Alexa Fluor^® ^488/CD25PE/CD4 Per CP) with 100 μl of cell suspension l antibody for 20 minutes followed by fixing with FoxP3 fix/perm buffer (Biolegend Cat. No. 421401) at room temperature for 20 minutes. The cells were then permeabilized in FoxP3 perm buffer (Biolegend Cat No. 421402,) for 20 minutes, labeled with FoxP3 for 20 min, and washed twice with FoxP3 perm buffer. The cells were then labeled with anti-Helios (22f6) (Biolegend Cat. No. 137203 Alexa Fluor^® ^647) for 20 minutes at room temperature, centrifuged, washed twice, resuspended, and analyzed by flow cytometry. Lymphocyte and macrophage populations were defined by forward and sideward scatter, according to cell granularity and size [18]. 10,000 cells is the standard sample size in most flow cytometric analyses, so 50,000 is sufficient for our methods. We determined that 5 μl of antibody was sufficient to stain cells at that concentration. We were always staining in a small sample volume. Though this is our exclusive method there is no need to increase the sample size.

The results were expressed as the total number of cells of the given cell phenotype being reported divided by the total number of cells counted (to normalize slightly different counts between tubes) multiplied by 100,000 (to work with whole numbers, since the percentages were often small). The total cells analyzed in most cases were greater than 50,000.

### Statistical analysis

T-cell expression data (CD4, CD25, FoxP3, Helios) for spleen and thymus cells of the three treatment groups (controls, VIP KO, and VIP KO + VIP) were summarized by descriptive statistics: medians, minimums, and maximums. Three pair-wise comparisons were made using non-parametric Mann-Whitney tests: controls *vs*. VIP KO, controls *vs*. VIP KO + VIP, and VIP KO *vs*. VIP KO + VIP. To adjust for multiple comparisons, a *P*-value of less than 0.017 was regarded as statistically significant.

## Results

### Results

Compared to WT mice, VIP KO mice had high numbers of cells expressing CD25, the IL-2 receptor α- chain. These CD25+CD4- thymic cells are in the lymphocyte gate. Treatment of the VIP KO mice with VIP suppressed these cells. In wild-type mice, the mean number of Treg cells was higher than that in untreated VIP KO mice, which showed a trend toward fewer Treg cells with CD4^+^CD25^+^FoxP3^+ ^in the spleen. Treatment with VIP increased the number of Treg cells with FoxP3^+^Helios^+^CD4^+^CD25^+ ^in spleen. Treg were induced intrasplenically in those cells CD4+CD25+FoxP3+Helios- since Helios is found on 100% of thymic-derived Treg and only 70% of splenic-derived Treg.^9 ^Therefore, lack of Helios in these VIP-induced Treg isolated from spleen indicates that they were generated from VIP interactions in spleen rather than migrating from the thymus. VIP KO mice also had more Treg lacking expression of FoxP3, the critical survival factor for Treg, compared to wild-type mice.

### Increased CD25+CD4-Cells in Thymus in VIP KO Mice: reduction with VIP treatment

In VIP KO mice, we identified CD25^+ ^(IL-2 receptor positive) cells which were CD4 negative. CD4^-^CD25^+^Helios^+ ^cells were markedly increased in the thymus of VIP KO mice (2689 cells, range 2520-2966) compared to control animals (3.0, range 0-22). They were reduced with VIP treatment, supporting the concept of VIP as an endogenous anti-inflammatory agent. VIP treatment of VIP KO mice reduced CD4^-^CD25^+^Helios^+ ^thymus cell numbers to 1558 with a range between 450-1783 (Table 1).

**Table 1:** Comparisons of T-cell expression in thymus cells among three mice groups

	Group 1: Controls (n = 10)		Group 2: VIP KO (n = 5)		Group 3: VIP KO + VIP (n = 5)		p-values for Group Comparisons		
	**Median**	**Min, Max**	**Median**	**Min, Max**	**Median**	**Min, Max**	**1 vs. 2**	**1 vs. 3**	**2 vs. 3**

**CD4-CD25+**	1146	666, 1831	3298	2953, 3517	3182	2579, 4109	0.0022*	0.0022*	0.47

**CD4-CD25+Helios+**	308	110, 436	2690	2520, 2966	1558	450, 1783	0.0022*	0.0022*	0.0091*

**CD4-CD25+FoxP3+Helios+**	3	0, 22	11	2, 15	43	21, 1771	0.18	0.0031*	0.0091*

**CD4+CD25+** **(Treg)**	159	56, 563	204	177, 281	432	377, 596	0.63	0.015*	0.0091*

**CD4+CD25+FoxP3+Helios-** **(Treg induced intrasplenically)**	44	0, 493	25	15, 58	48	25, 493	0.81	0.63	0.12

**CD4+CD25+FoxP3-Helios+** **(Treg)**	46	4, 79	141	111, 166	141	23, 171	0.0022*	0.028	0.92

**CD4+CD25+FoxP3+Helios+** **(Treg)**	21	0, 423	25	15, 53	31	16, 378	0.81	0.40	0.26

Note: p-values were based on Mann-Whitney tests; *: statistically significant at adjusted alpha = 0.017.

### Increased numbers of FoxP3-Treg in Thymus in VIP KO Mice

VIP KO mice, compared to wild-type mice, had increased mean numbers (141 *vs*. 46) of Treg cells lacking FoxP3 expression (CD4+CD25+FoxP3-Helios+).

### Increased Treg with VIP therapy in thymus of VIP KO mice

**Figure 1:**
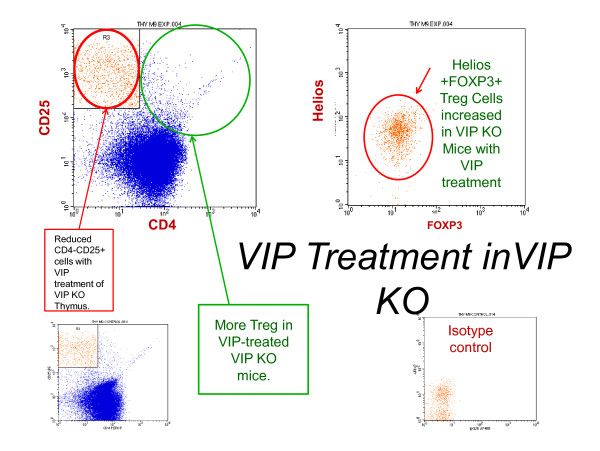
**VIP Facilitates Thymic Production Of Treg (Helios+Foxp3+CD4+CD25+)**. In this VIP KO mouse thymus treated with VIP, there were 596.09 Tregs expressing CD4 and CD25. This is in contrast to untreated VIP KO mice (fig. 3) which is associated with fewer Treg. Gated on CD4-CD25+ lymphocytes (which were orange cellsin the figure above, there are more Helios+CD25+FoxP3+CD4- cells in VIP-treated VIP KO mice than seen in untreated VIP KOmice shown in Fig.2 (red circle with arrow).

**Figure 2:**
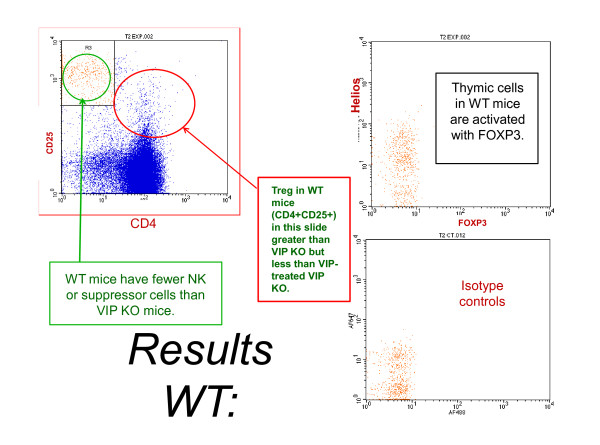
**Wild-Type Thymus Associated With Lower Treg Numbers Than VIP-Treated VIP KO MiceA control mouse had intermediate levels of CD4+CD25+ thymic Tregs (384**.14) compared to VIPKO (177.07) and VIP-treated VIP KO (596.09). This supports the concept that VIP can increase Treg centrally in thymus. Wild-type mice have fewer CD25+ cells compared VIP KO, suggesting that VIP is critical for suppression of CD25+ cell expression.

To determine if absence of VIP leads to suppression of regulatory T cells, we quantified Treg in thymus and spleen from control and VIP KO mice. In the thymus, VIP KO mice had Tregs (CD4^+^CD25^+^) levels similar to control mice, (204 cells; range 177-281, *vs*. 159; range 56-563), which were increased with VIP treatment, with *P *= 0.0091 (Table 1). An example of a VIP KO mouse treated with VIP leading to high numbers of thymic Treg compared to an untreated VIP KO mouse is shown in Figure 1. Also in Figure 2., VIP treatment elevates Treg numbers at high levels even greater than untreated wild-type.

Control and VIP KO spleen levels of Treg (triple-staining CD4^+^CD25^+^FoxP3^+^) cells were statistically similar, yet showed a trend towards suppression in VIP KO mice: 109, range 8-359 for the wild-type, and 9, range 4-37 for VIP KO. Treatment with VIP increased these Treg (triple-staining) cells in VIP KO mice. Treatment with VIP in VIP KO mice increased triple-staining Treg (CD4^+^CD25^+^FoxP3^+^) cell levels from 9.2 (range 4-37) in untreated VIP KO to 150 (range 99-226) in VIP-treated VIP KO mice; this was statistically significant (*P *= 0.0091). Quadruple-staining CD4^+^CD25^+^FoxP3^+^Helios^+^, Treg cell numbers were similar in control (68 cells, range 2-251) and VIP KO mice 6, range 0-22). Treatment with VIP in VIP KO mice resulted in increased Treg (quadruple-staining CD4^+^CD25^+^FoxP3^+^Helios^+^) cells (82; range 53-141), which was statistically significant *P *= 0.0091.

With our small sample size, there was a trend, though lack of statistical baseline suppression, in Treg in thymus and spleen in VIP KO mice. It is, however, evident that a statistically significant milieu exists with CD25^+^CD4^- ^cells, and that VIP not only is able to suppress these CD25^+^CD4^- ^cells, but also is simultaneously able to increase Treg, expressing triple and quadruple-staining CD4^+^CD25^+^FoxP3^+ ^and CD4^+^CD25^+^FoxP3^+^Helios^+ ^cells.

Tables 1 and 2 summarize spleen and thymus data among the three groups: 1) control; 2) VIP KO; and 3) VIP KO+VIP. Centrally, in the thymus of VIP KO mice, CD25^+^CD4^- ^cell numbers were high--3297 cells (median) with a range from 2952 to 3517--whereas control C57BL/6 mice numbers were lower: 1145 (range 665-1830), supporting a pro-inflammatory milieu in the absence of the VIP gene (*P *= 0.0022). When untreated VIP KO mice thymus CD25^+^CD4^-^Helios^+ ^cells were compared to VIP-treated VIP KO, the levels (2689, range 2519-2966) were suppressed to 1558 (range 450-1783), *P *= 0.0091.

**Table 2:** Comparisons of T-cell expression in spleen cells among three mice groups

	Group 1: Controls (n = 10)		Group 2: VIP KO (n = 5)		Group 3: VIP KO + VIP (n = 5)		p-values for Group Comparisons		
	**Median**	**Min, Max**	**Median**	**Min, Max**	**Median**	**Min, Max**	**1 vs. 2**	**1 vs. 3**	**2 vs. 3**

**CD4-CD25+**	29	6, 73	31	19, 429	47	33, 89	0.63	0.12	0.18

**CD4-CD25+Helios+**	1	0, 4	4	0, 164	2	0, 10	0.046	0.31	0.61

**CD4-CD25+FoxP3+Helios+**	4	0, 19	0	0, 0	0	0, 2	0.069	0.32	0.14

**CD4+CD25+** **(Treg)**	435	88, 1383	374	199, 611	380	198, 489	1.00	1.00	0.61

**CD4+CD25+FoxP3+Helios-** **(Treg induced intrasplenically)**	109	8, 359	9	4, 37	150	99, 226	0.038	0.91	0.0091*

**CD4+CD25+FoxP3-Helios+** **(Treg)**	83	0, 270	254	108, 352	96	21, 157	0.028	0.91	0.029

**CD4+CD25+ FoxP3+Helios+** **(Treg)**	68	2, 251	6	0, 22	82	53, 141	0.087	0.91	0.0091*

Note: p-values were based on Mann-Whitney tests; *: statistically significant at adjusted alpha = 0.017.

### Treg Induced peripherally in spleen in VIP KO Mice treated with VIP

**Figure 3:**
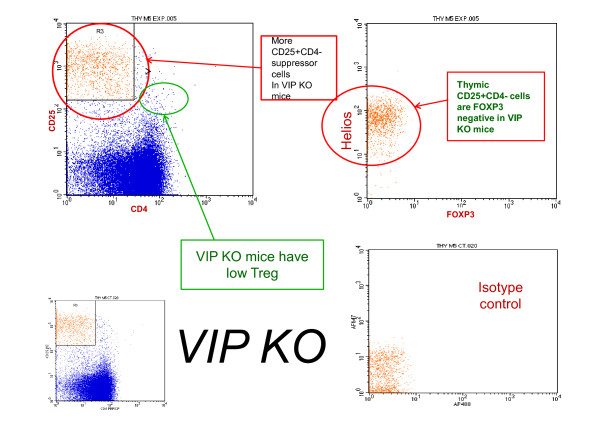
**Lack Of VIP In VIP KO Mice Associated With Trend Towards Low Tregs in spleen or thymus? (Heliosfoxp3+CD4+Cd25+)**. In an untreated VIP KO mouse, thymus CD4+CD25+ cells were less plentiful (177.07) compared to the 596.09 seen in the VIP-treated VIP KO mouse above. Gated on CD4-CD25+ lymphocytes, only 15 Helios+CD25+FoxP3+CD4- cells were detected.

Peripherally, in spleen, untreated VIP KO mice numbers of Treg (CD4^+^CD25^+^FoxP3^+^) cells were 9.2 (range 3.6-36), whereas VIP-treated VIP KO mice increased levels to 150.4 (range 98-226). *P *= 0.0091. Treg (Quadruple-staining CD4^+^CD25^+^FoxP3^+^Helios^+^) cells were low in untreated VIP KO mice (5, range 0-21), and the level increased with VIP-therapy to 82 (range 52-140), *P *= 0.0091. These are peripherally-induced Treg in spleen since they lack Helios which is found in 100% of thymic Treg and only 70% of splenic Treg. Absence of Helios in these samples obtained from spleen indicates intrasplenic activation. An example of a VIP KO mouse with low numbers of Treg is shown in Figure 3.

## Discussion

This study demonstrates that VIP KO mice have spontaneous accumulation of CD25^+^CD4^- ^cells in the central thymus compartment compared to wild-type mice. These levels decrease in VIP KO mice treated with VIP. Treg lacking FoxP3 were more plentiful in VIP KO mice compared to wild-type, supporting the concept that VIP is critical for immune tolerance.

Likewise, we showed decreased Treg centrally in thymus (CD25+CD4+Helios+ cells) and a trend towards reduction in immune tolerant CD4^+^CD25^+^FoxP3^+ ^Treg cells in VIP KO mice. Pituitary Adenylate Cyclase Activating Peptide (PACAP), a peptide similar to VIP in sequence and function, has recently been reported to enhance production of Treg [19]. So, since our VIP KO mice were not double knockouts for VIP along with PACAP, it is plausible that PACAP is the reason for residual Treg. Nevertheless, suggesting a more dominant role of VIP versus PACAP, this trend towards decreased Treg was present in VIP KO mice *vs*. wide variance among wild-type mice, using the most conservative statistical adjustments.

VIP treatment of VIP KO mice was, nonetheless, statistically significant in increasing Treg numbers (CD4^+^CD25^+^FoxP3^+^Helios^+^) and suppressing a pro-inflammatory cell phenotype (CD25^+^CD4^-^). We did not determine the origin of these CD4+CD25+FoxP3+Helios+ cells--*i.e*., from the thymus or originating intra-splenically. Statistically significant increases in thymus Treg (CD4+CD25+) and spleen Treg (CD4+CD25+FoxP3+) were seen in VIP-treated VIP KO mice compared to untreated VIP KO mice, supporting the concept that VIP treatment induces the generation of Treg. Indeed, in other mouse models VIP induces the generation of these Treg from the CD4^+^CD25^- ^T cell compartment.[20] Treg isolated from spleen have an intrasplenic origin since they lack Helios, suggesting that VIP acts peripherally, directly on the spleen. This is a novel finding not studied during the creation of the VIP KO mouse model[21] in 2003, though supported by subsequent literature postulating therapeutic potential for immune homeostasis in 2007.[17]

The goal of this paper was to explore the development of T regulatory cells in VIP knockout mice, particularly in the thymus and spleen, and support the concept that exogenous VIP is able to induce Tregs. Future studies we are contemplating include: 1) treating VIP KO animals for 2 weeks (15 ng VIP qod for 7 doses), followed by a 1 month hiatus and then analyzing treg populations in spleen in thymus--to determine if there are long-term effects on Treg with VIP treatment; 2) *In vitro *stimulation of Tregs from VIP KO mice to show their Helios and FoxP3 expression by isolating naïve CD4+CD25- cells from thymus and spleen and stimulating them for 5 days with CD3/CD28 beads and TGF Beta and IL-2. Expression of CD4, CD25, FoxP3 and Helios by FACS will subsequently by determined--this will verify if absence of VIP in Tregs affects expression of these markers.

Our data support those reported by Delgado *et al*. on the emerging, mounting evidence of the essential role of VIP in immune tolerance, with particular modulation of Treg. Tolerance to antigen is essential to alleviating clinical manifestations of allergic diseases such as allergic rhinitis, allergic asthma, and stinging insect anaphylaxis. Autoimmune diseases such as Systemic Lupus Erythematosus (SLE) are the epitome of self versus non-self disarray, and knowing that FoxP3^+ ^Treg cells are depleted during attacks and flares of lupus, supports the concept of VIP's potential as a drug for SLE and other autoimmune diseases and disorders with vasculitis.

It is possible that the CD4-CD25+ cells are not inflammatory lymphocytes since they could be CD8+CD25+ effector cells. Future experiments will be directed at determining this phenotype. Although we showed that VIP KO mice have Treg lacking FoxP3 (CD4+CD25+FoxP3-), they may be activated effector cells; yet, the absence of FoxP3 would suggest that they lack a critical molecule necessary for Treg survival.

In summary, lack of VIP in mice leads to CD25+CD4- cells; VIP treatment suppresses these cells and increases Treg in spleen. These experiments support the dual role of VIP as an anti-inflammatory agent and immune tolerance promoter.

## Competing interests

The authors declare that they have no competing interests.

## Authors' contributions

AS conceived the study and carried out the surgery and flow cytometry experiments, TR ran the FACSscan machine, MG assisted in analysis of flow data, SH participated in design and coordination, JC conducted statistical analysis. All authors have read and approved the final manuscript.
